# Mycelial pellet formation by edible ascomycete filamentous fungi, *Neurospora intermedia*

**DOI:** 10.1186/s13568-016-0203-2

**Published:** 2016-04-22

**Authors:** Ramkumar B. Nair, Patrik R. Lennartsson, Mohammad J. Taherzadeh

**Affiliations:** Swedish Centre for Resource Recovery, University of Borås, 50190 Borås, Sweden

**Keywords:** *Neurospora intermedia*, Ascomycete, Filamentous fungi, Edible fungi, Pellet

## Abstract

Pellet formation of filamentous fungi in submerged culture is an imperative topic of fermentation research. In this study, we report for the first time the growth of filamentous ascomycete fungus, *Neurospora intermedia* in its mycelial pellet form. In submerged culture, the growth morphology of the fungus was successfully manipulated into growing as pellets by modifying various cultivation conditions. Factors such as pH (2.0–10.0), agitation rate (100–150 rpm), carbon source (glucose, arabinose, sucrose, and galactose), the presence of additive agents (glycerol and calcium chloride) and trace metals were investigated for their effect on the pellet formation. Of the various factors screened, uniform pellets were formed only at pH range 3.0–4.0, signifying it as the most influential factor for *N. intermedia* pellet formation. The average pellet size ranged from 2.38 ± 0.12 to 2.86 ± 0.38 mm. The pellet formation remained unaffected by the inoculum type used and its size showed an inverse correlation with the agitation rate of the culture. Efficient glucose utilization was observed with fungal pellets, as opposed to the freely suspended mycelium, proving its viability for fast-fermentation processes. Scale up of the pelletization process was also carried out in bench-scale airlift and bubble column reactors (4.5 L).

## Introduction

Filamentous fungi are widely used industrial microorganisms that contribute to the global economy via production of a plethora of important products such as antibiotics, enzymes, organic acids, human/animal food or pharmaceutical product (Liao et al. 2007; Prajapati et al. 2014; Zhang and Zhang 2015). The ascomycete filamentous fungi *Neurospora intermedia* studied in this work has been traditionally used for the preparation of an indigenous Indonesian food, oncom. Hence, the fungus is classified as ‘edible’ and categorized as ‘generally regarded as safe’ (GRAS). *N. intermedia* also possess high ethanol fermenting capability compared to other edible filamentous fungi such as *Aspergillus oryzae,Fusarium venenatum,Monascus purpureus, Rhizopus* sp. (Ferreira et al. 2014). With its high protein content (56 % w/w) and the potential of utilizing pentose sugars, *N. intermedia* has potential application in production of ethanol and protein-rich fungal biomass (for fish or animal feed), from substrates such as stillage from wheat-based ethanol industries (Bátori et al. 2015) (Ferreira et al. 2015) or lignocellulose waste (Nair et al. 2015). However, due to its filamentous growth, the cultivation of this filamentous fungus in large scale industrial bioreactors can be a challenge.

A potential solution to overcome the problems associated with the filamentous growth is to obtain growth in the form of pellets. Filamentous fungi can be grown in submerged cultures in several different morphological forms such as free suspended mycelia (with typical diameters of 2–18 µm), or as mycelial clumps or as pellets (Liao et al. 2007; Ward 2012). Pellet formation of the filamentous fungi in submerged cultivation has attracted the attention of researchers and industrial engineers since decades. Pellet morphologies have been classified into three different groups encompassing, (a) pellets with a compact central core and a fluffy (hairy) or loosely packed filamentous outer zone; (b) with a compact core that is smooth with limited lateral growth and; (c) compact pellets with a hollow core (Cox and Thomas 1992). Growth in the form of pellets has previously been reported in many filamentous fungal species such as the most studied *Aspergillus*, *Rhizopus* or *Penicillium* strains (Fujita et al. 1994; Liu et al. 2008; Saraswathy and Hallberg 2005; Zhou et al. 2000). However, *N. intermedia* growth in the form of pellets is not reported in the literature. Fungal growth in the form of pellets possesses several potential advantages such as ease of biomass harvesting, the low viscosity of the fermentation medium, improved oxygen diffusion and high yield of products (Hille et al. 2009; Zhang and Zhang 2015). It has been suggested that pellets are formed as a result of the influence of many cultivation factors through a complex interaction process. These factors include the inoculum size, pH, dissolved oxygen level, agitation, nucleating agents, additives, trace metals, temperature, reactor types, etc. (Junker 2006; Wargenau et al. 2013; Wösten et al. 2013; Zhang and Zhang 2015). However, these factors are greatly depended on the microbial strain and the specific cultivation conditions used. Each factor has varying effects on the growth morphologies of different fungal species. For example, pellet formation in several strains of *Rhizopus* sp. are influenced by trace metals (Zhou et al. 2000), inoculum size (Liao et al. 2007), agitation (Liu et al. 2008), Ca^2+^ concentration, pH and temperature (Nyman et al. 2013). Strains of *Penicillium chrysogenum* require high pH to form pellets (Metz and Kossen 1977), while carbon sources play a major role in pellet formation of *Aspergillusterreus* (Jia et al. 2009). Hence, the study on fungal pellet formation is limited specifically to individual fungal species.

In this paper, we report for the first time the growth of the edible ascomycetes filamentous fungi, *N. intermedia* as pellets. Factors such as pH, carbon source, additive agents, trace metals and agitation that generally influence the fungal pelletization process are studied for *N. intermedia* pellet formation. Scaling up the process in a bench-scale airlift and bubble column reactors (4.5 L), were also carried out in this study to determine the stability of pelletization process at higher cultivation volume.

## Materials and methods

### Fungal strain

An edible ascomycete, *Neurospora intermedia* CBS 131.92 (Centraalbureau voor Schimmelcultures, Netherlands), was maintained on potato dextrose agar (PDA) slants containing (in g/L): potato extract 4, d-glucose 20, agar 15 and the slants were renewed every 6 months. For the regular experimental purpose, the fungus was transferred to fresh PDA plates containing (in g/L): potato extract 4, d-glucose 20 and agar 15. The fungal plates were then incubated aerobically for 3–5 days at 30 °C. For preparing spore suspension, fungal plates were flooded with 20 mL sterile distilled water and the spores were released by gently agitating the mycelium with a disposable cell spreader. An inoculum of 50 mL spore suspension per L medium with a spore concentration of 6.3 ± 0.8 × 10^5^ spores/mL was used for the cultivations.

### Standard cultivation

The fungal cultures were carried out aerobically in a liquid semi-synthetic potato dextrose medium (containing 20 g/L glucose and 4 g/L potato extract), unless otherwise specified. Cultivations were made in 100 mL volume (in 250 ml Erlenmeyer flasks), for 72 h in an orbital shaking water bath (Grant OLS-Aqua pro, UK) at 35 °C and 125 rpm (with an orbital shaking radius of 9 mm and a flask diameter of 85 mm), with samples taken every 24 h, unless otherwise specified. The pH was adjusted with either 2 M HCl or 2 M NaOH. All experiments and analyzes were carried out in duplicate and results reported with error bars and intervals representing two standard deviations.

### Pelletization in shake flask: screening of factors

Based on preliminary results (data not shown), factors such as pH, cultivation agitation rate, type of the carbon source, the presence of additive agents and trace metals were screened separately for their effects on *N. intermedia* pellet formation. A defined synthetic medium with (g/L) d-glucose (or another carbon source) 20, NH_4_Cl 7.5, MgSO_4_·7H_2_O 0.5, NaCl 1.0, and KH_2_PO_4_ 3.5 was used as the basal screening medium for the pellet formation. pH 5.5 (standard optimum value) was used for all the screening cultures, unless otherwise specified.

The pH of the cultivation medium at six levels (pH 2.0, 4.0, 5.5, 7.0, 9.0 and 10.0) and agitation rate at three levels (100, 125, and 150 rpm) were screened. The carbon source (20 g/L) screened was glucose, arabinose, sucrose, and galactose. The additive agents used were glycerol (2 % v/v) and calcium chloride (2 g/L basal screening medium). A trace metal solution (Sues et al. 2005), at the concentration 15 mL/L basal screening medium was used to study the effect of trace metals on pellet formation.

### Cultivation in acidic pH conditions

The effect of acidic pH of the culture media on *N. intermedia* pellet formation was determined. The fungus was grown aerobically at different acidic pH conditions such as 3, 3.5, 4 and 4.5, in potato dextrose medium. pH of the medium was adjusted initially by the addition of 2 M HCl. Cultivation at pH 5.5 served as control. To determine the independent effect of agitation on pellet morphology at acidic conditions (pH 3.0), three test runs were made with cultivations carried out at different agitation rates: 100, 125 and 150 rpm with an orbital shaking radius of 9 mm and a flask diameter of 85 mm.

### Scale up of the pellet formation

The fungal pellet formation was investigated in a 4.5 L airlift bioreactor (Belach Bioteknik, Stockholm, Sweden) with a working volume of 3.5 L and an aeration rate of 1.42 vvm (volume_air_/volume_media_/min). Pellet formation was also investigated in a bubble column reactor (achieved by removing the internal loop of the existing airlift reactor) with the working volume of 3.5 L and at a reduced aeration rate (maintained at 0.71 vvm), compared to the airlift cultivation. An air-sparger with a pore size of 90 µm was used for aeration in both airlift and bubble column cultures. Potato dextrose medium, with its pH initially adjusted to 3.0 ± 0.5 using 2 M HCl was used for the cultivation. The temperature was maintained at 35.0 ± 0.5 °C throughout the cultivation and samples were collected every 24 h.

### Analytical methods

Initial spore concentration was measured using a Bürker counting chamber (with a depth of 0.1 mm) under a light microscope (Carl Zeiss Axiostar plus, Oberkochen, Germany). The spore solution was diluted ten times before the measurement, and the spores were counted in a volume of 1/250 μl each. HPLC (Waters 2695, Waters Corporation, Milford, USA) was used to analyze all liquid fractions. A hydrogen-based ion-exchange column (Aminex HPX-87H, Bio-Rad Hercules, CA, USA) at 60 °C with a Micro-Guard cation-H guard column (Bio-Rad) and 0.6 mL/min 5 mM H_2_SO_4_, as eluent, was used for the analyzes of glucose, ethanol, glycerol and acetic acid. A UV absorbance detector (Waters 2487), operating at 210 nm wavelength, was used in series with a refractive index (RI) detector (Waters 2414). The morphology of the fungal biomass was determined by visual examination of the 72 h submerged culture (mycelium or pellets). Fungal biomass concentration (dry weight) was determined at the end of the cultivation by washing the pellet or mycelial biomass with deionized water followed by drying at 105 °C for 24 h before weighing. A digital Vernier caliper (Limit, Alingsås, Sweden) with a resolution of 0.01 mm was used to measure the pellet diameter. An average of 50–100 pellets (from shake flask and bench-scale reactor culture, respectively) was measured for the pellet size determination.

### Statistical analysis

Statistical analysis of the data was carried out using MINITAB^®^ 17 (Minitab Inc., State College, PA, USA) software. A box-and-whisker plot was developed to assess and compare the fungal pellet size distribution. The box plot represents the pellet-size distribution graph with the first quartile Q1 (with 25 % of the data values), the median (with half of the data values) and the third quartile Q3 (with 75 % of the data values), with pellet diameter obtained at varying culture conditions (agitation) as the major variable. By default the upper and lower whiskers extend to the highest and lowest data value (among the distribution), respectively. A MINITAB^®^ 17 generated histogram, with a Gaussian curve maximum was used to assess the size distribution and the average pellet diameter from the bench-scale reactors. A sample size of 50 or 100 pellets, from the shake flask or bench-scale reactor culture (respectively), was measured to obtain the pellet size distribution.

## Results

Growth patterns of certain filamentous fungal species such as *Aspergillus*, *Rhizopus* or *Penicillium* strains can be manipulated to form mycelial pellets in submerged cultures, and it had been studied extensively. The edible ascomycete filamentous fungus *N. intermedia*, studied in this work has not been reported to grow as pellets, to the best of our knowledge. In this study, the fungus was successfully manipulated into growing as pellets for the first time. Five different factors as pH (2.0, 4.0, 5.5, 7.0, 9.0 and 10.0), agitation rate (100, 125 and 150 rpm), carbon source (glucose, arabinose, sucrose and galactose), presence of additive agents (glycerol and calcium chloride) and trace metals were investigated for their effect on the pelletization process. The direct influence of acidic pH on the fungal morphology, with uniform mycelial pellets formed in the pH range 3.0–4.0 was observed. In a modified fed-batch cultures with sequential addition of glucose, stable pellet morphology was observed throughout the fermentation period (192 h). The pelletization process was unaffected by the type of inoculum used; either in the form of fungal spores, mycelial clump or pellets. Scaling-up of the process in airlift and bubble column reactors showed that the pelletization process was stable over the aeration rates and the reactor models. With the previous reports on the ethanol production potential of the fungal strain, ethanol fermenting capabilities of the *N. intermedia* pellets were also investigated. It was observed that the fungal pellet cultures showed relatively higher ethanol yield compared to the mycelial clump form, at similar conditions. Screening of the factors favoring pellet formation with the detailed study of the *N. intermedia* pelletization process is reported in the following sections.

### Pellet formation

From the five different factors screened (at various levels) which included pH, carbon source, agitation, presence of additive agents, and trace metals (Table 1); pH of the media was identified to be the most influential factor for the pellet formation. Small fungal pellets were observed at pH 4.0 with a yield of 0.118 g/g dry fungal biomass and 0.366 g/g ethanol per initial glucose consumed (Table 1). The fungal growth was observed in all conditions, except pH 2.0, with a lowest yield of 0.118 g/g dry biomass per carbon source consumed (observed with small fungal pellets at pH 4.0) to a maximum of 0.452 g/g dry biomass per carbon source consumed (observed with pH 9.0) (Table 1). The different factors screened, resulted in a fungal growth ranging from large spherical pellets to completely dispersed mycelia. Particularly, the fungal morphology could be grouped into either (a) thick mycelial clumps (aggregated or entangled filamentous form of the fungus) as observed in control cultivation; (b) loose or freely suspended mycelia as observed with cultivations in presence of additives (such as glycerol and calcium ions); and (c) pellets as observed at acidic pH cultivation conditions. The choice of carbon source did not show any significant influence on the type of fungal growth. In all cultures with different carbon sources (at an initial culture pH of 5.5), growth in the form of mycelial clumps was observed. Biomass yields in the range from 0.184 g/g (with galactose) to 0.440 g/g (with arabinose) dry fungal biomass from initial substrate consumed were detected. However, the use of the additives glycerol and calcium chloride (with an initial culture pH of 5.5), resulted in the formation of loose mycelial filaments with a biomass yield of 0.280 and 0.323 g/g glucose consumed, respectively. From all tested agitation levels (with an initial culture pH of 5.5), only freely suspended mycelium were observed. The biomass yield ranged from 0.206 to 0.331 g/g glucose consumed. Ethanol and glycerol were the major metabolites produced with a yield from 0.140 to 0.366 g and 0.007 to 0.031 g/g substrate consumed, respectively (Table 1).

**Table 1:** Fungal biomass and ethanol production at various cultivation conditions

Factors	Levels	Morphology	Y_E/S_^b^ (g/g)	Y_B/S_^a^ (g/g)	Y_Gly/S_^a^ (g/g)
pH	10.0	Loose filaments	ND	0.435	0.032
	9.0	Filamentous	ND	0.452	0.031
	7.0	Filamentous	0.192	0.279	0.014
	5.5 (control)	Filamentous	0.205	0.262	0.016
	4.0	Pellets	0.367	0.118	0.008
	2.0	ND	ND	ND	ND
Additive agents	Glycerol	Loose filaments	0.193	0.280	0.012
	Calcium ions (CaCl_2_∙2H_2_O)	Loose filaments	0.140	0.323	0.016
Carbon source	Sucrose	Filamentous	0.195	0.284	0.009
	Arabinose	Filamentous	0.000	0.440	0.027
	Glucose	Filamentous	0.217	0.250	0.016
	Galactose	Filamentous	0.295	0.184	0.012
Trace metals		Filamentous	0.214	0.261	0.012
Agitation	100 rpm	Filamentous	0.266	0.206	0.014
	120 rpm	Filamentous	0.213	0.256	0.012
	150 rpm	Filamentous	0.143	0.331	0.017

*ND* not detected

^a^Maximum ethanol yield on substrate consumed

^b^Maximum biomass yield on substrate consumed

^c^Maximum glycerol yield on substrate consumed

### Acidic pH and pellet formation

**Fig. 1:**
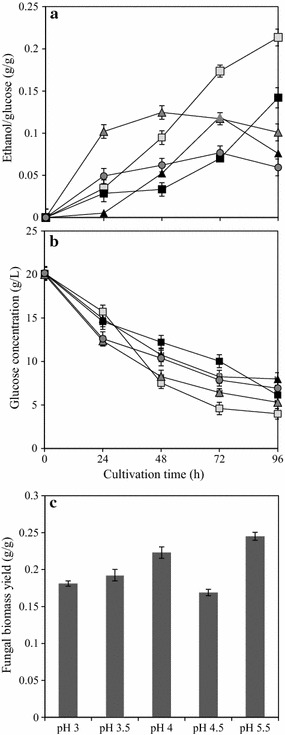
Pellet formation at varying pH conditions **a** Ethanol yield and **b** glucose consumption at pH 3.0 (*filled square*), 3.5 (*filled square*) 4.0 (*filled triangle*), 4.5 (*filled triangle*) and control at pH (5.5) (*filled circle*) and **c** fungal biomass yield (g dry fungal biomass/g glucose consumed) at varying pH conditions

In the previous section, acidic pH conditions favored pellet formation. Hence, the influence of pH was further studied in the range of 3.0–5.5. The best pellet formation was found to be at pH 3.0–3.5 with distinctly uniform and separated pellets (2–3 mm in diameter). At pH 3.0, the pellets were smaller (with an average diameter of 2.38 ± 0.12 mm) than at pH 3.5 (with an average diameter of 2.86 ± 0.38 mm). Non-uniform pellet size distribution was observed at pH 4.0, with an average diameter of 3.05 ± 0.91 mm and a reduced final biomass concentration to 0.16 g/g dry fungal biomass from initial glucose consumed. At varying pH conditions the final biomass concentration ranged between 0.18 g/g (observed at pH 3) to 0.24 g/g (at pH 5.5) dry fungal biomass per initial glucose consumed. However, biomass growth in the form of freely suspended mycelia and filamentous mycelial clump was observed at pH 4.5 and 5.5 (control) respectively. Hence at pH above 4, filamentous growth in the form of mycelial clump was most favored, with a reduced biomass yield. The biomass yield however increased further at the optimum pH conditions, 5.5 (Fig. 1). A distinct correlation between fungal pellet morphology at lower pH and metabolite production was observed (Fig. 1). Ethanol production with the range 0.26 g/g (at pH 3.0) to 0.11 g/g (at pH 5.5) per initial glucose consumed was observed (with 56 % reduction in the yield). However, the glycerol concentrations remain negligible at lower pH conditions, with its increase to a maximum concentration of 0.021 g/g glucose consumed (at pH 5.5).

**Fig. 2:**
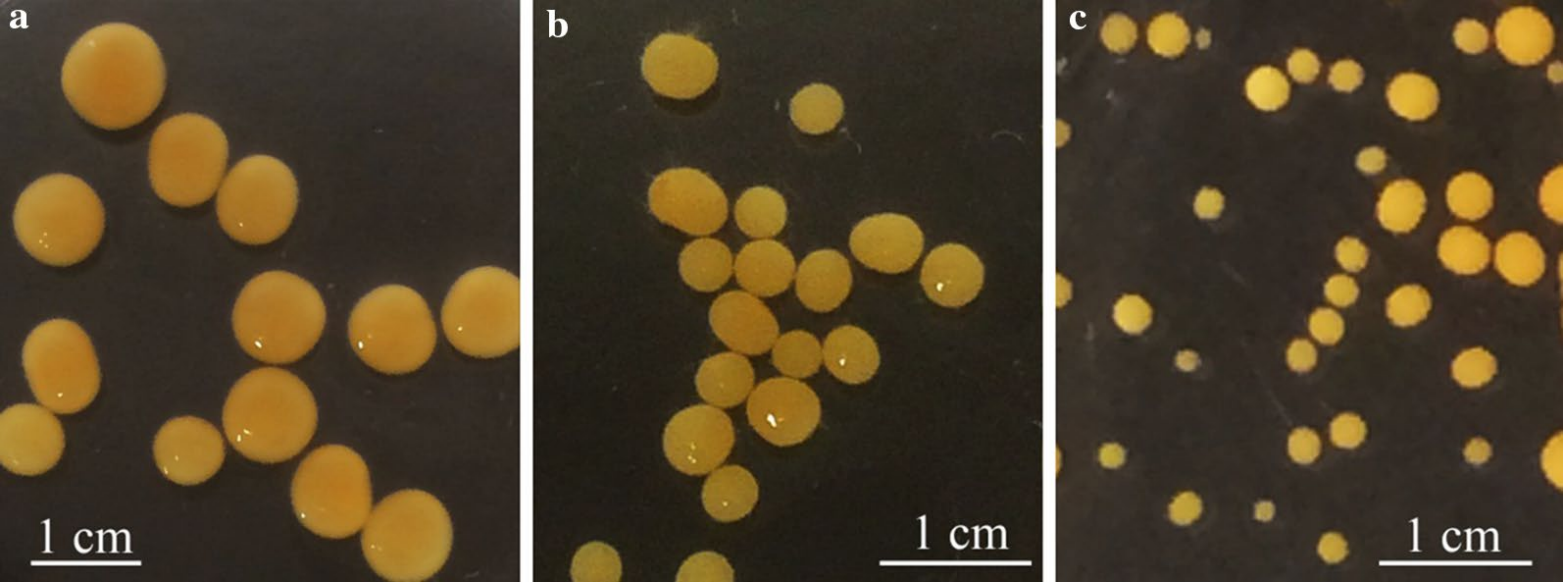
Variation in fungal pellet size at varying agitation rates **a** 100 rpm **b** or 125 rpm and **c** 150 rpm at culture pH 3 (with an orbital shaking radius of 9 mm and a flask diameter of 85 mm)

**Fig. 3:**
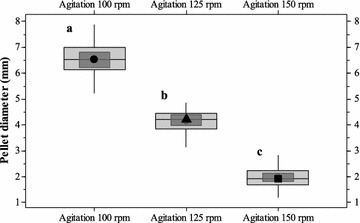
Box-and-whisker plot for the size distribution of fungal pellets at varying agitation rates: 100 rpm (*filled circle*), 125 rpm (*filled triangle*) and 150 rpm (*filled square*) (with an orbital shaking radius of 9 mm and a flask diameter of 85 mm) at the culture pH 3. Values in the *graph* represent mean values (diameter in mm) with; **a** First quartile 6.15, median 6.54, third quartile 7.01, whiskers 5.24 (*lower*)–7.88 (*upper*) and 95 % confidence interval for the median in range: 6.21–6.83 for agitation 100 rpm; **b** first quartile 3.85, median 4.22, third quartile 4.45, whiskers 3.14 (*lower*); 4.86 (*upper*) and 95 % confidence interval for the median in range: 3.14–4.86 for agitation 125 rpm; **c** first quartile 1.69, median 1.92, third quartile 2.24, whiskers 1.21 (*lower*); 2.84 (*upper*) and 95 % confidence interval for the median in range: 1.81–2.14 for agitation 150 rpm

Cultivations carried out at pH 3.0, with varying agitation rates (100, 125 and 150 rpm) showed no significant influence of the agitation rates on the *N. intermedia* pellet formation. Pellets were formed in all the experimental runs irrespective of the agitation rates of the cultivation (Fig. 2). However, the agitation rate influenced the size distribution of the pellets, with an average pellet diameter of 6.54 ± 0.62, 4.22 ± 0.41 and 1.92 ± 0.33 mm at 100, 125 and 150 rpm agitation, respectively (Fig. 3). Furthermore, at lower agitation rates a reduction in the glucose utilization was observed at the end of cultivation. Fungal biomass concentration 0.241, 0.225 and 0.303 g/g dry fungal biomass from initial glucose consumed with a maximum ethanol concentration of 0.232 ± 0.1, 0.259 ± 0.2, and 0.178 g ethanol/g glucose consumed was observed at agitation rate 100, 125 and 150 rpm respectively.

### Pellet formation by increased glucose concentration

**Fig. 4:**
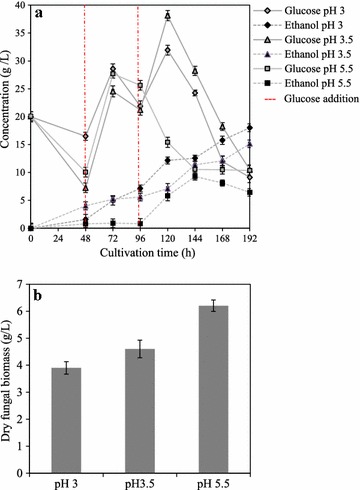
Pellet growth at increasing glucose concentrations. **a** Glucose assimilation (*solid line*) with subsequent ethanol production (*dotted line*) at pH 3.0 (*diamond*), 3.5 (*triangle*) and control pH 5.5 (*square*) and **b** fungal biomass at varying pH conditions during modified fed batch fermentation

Morphological stability of the fungal pellets was studied in a modified fed-batch fermentation method with the sequential addition of glucose substrate at the intervals of 48 h. The pellets formed during the initial 48 h of the culture, at the acidic conditions (pH 3.0 and 3.5), maintained their pellet morphology throughout the cultivation period (192 h). However, towards the end of the cultivation (144–192 h), short hairy filaments were observed on the pellet surface. At the end of the cultivation, a final biomass concentration of 3.9, 4.6 and 6.2 g/L dry fungal biomass was observed at pH 3.0, 3.5 and 5.5 respectively (Fig. 4). An increased pellet size at pH 3.5 (5.1 ± 0.8 mm) was observed, compared to pellets grown at pH 3.0 (2.9 ± 0.5 mm). A maximum ethanol production of 17.9 ± 0.7 and 15.5 ± 0.4 g/L was observed with fungal pellets at pH 3.0 and 3.5 respectively, as opposed to 9.3 ± 0.1 g/L ethanol from the freely suspended mycelium. The addition of glucose (at 96 h) to pellet cultures at pH 3.0 and 3.5 yielded 0.503 g and 0.494 g/g ethanol from glucose consumed within 24 h fermentation time, whereas freely suspended mycelium produced 0.165 g/g ethanol from glucose consumed. Hence, the results suggest that fungal pellets could be used effectively for fast-fermentation methods with efficient glucose utilization and increased ethanol production, as opposed to the freely suspended mycelium (Fig. 4).

### Effect of inoculation method on pellet formation

**Fig. 5:**
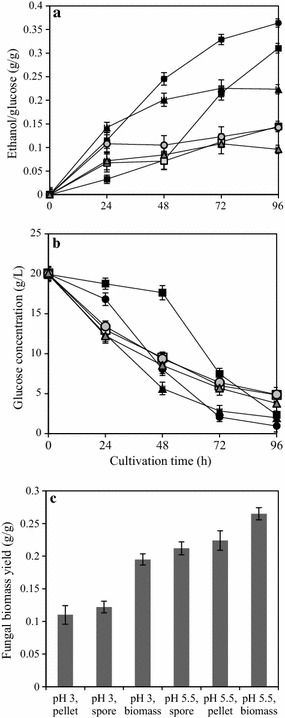
Effect of inoculation method on fungal pellet growth: **a** Ethanol yield and **b** glucose consumption at pH 3.0 with pellet (*filled circle*), spore (*filled square*) or biomass (*filled triangle*) inoculum, and at pH 5.5 with pellet (*filled circle*), spore (*filled square*) or biomass (*filled triangle*) inoculum and **c** fungal biomass yield (g dry fungal biomass/g glucose consumed) at varying pH conditions with different inoculation methods

Fungal pellet formation at acidic pH was studied further to strengthen the hypothesis that pelletization in *N. intermedia* is activated at acidic pH conditions, irrespective of the inoculation methods adopted. Pellet formation was observed at acidic pH 3.0 irrespective of the nature of the inoculum used, either fungal spores or mycelial clumps or pellets. In cultures (at pH 3.0) that used pellets as the inoculum source, smaller pellets (with average diameter 2.6 ± 0.2 mm) were formed. It was observed that the fungal pellets originally from the inoculum were significantly larger (7.4 ± 0.8 mm) in size at the end of the cultivation (96 h). Irrespective of the inoculum method adopted, fungal biomass yield of the pellets formed at pH 3.0 was lower than the control at pH 5.5. Biomass yields of 0.115, 0.122 and 0.195 g/g glucose consumed with ethanol yields of 0.38, 0.35, 0.26 g/g glucose consumed were observed at pH 3.0 with pellet, spore, or mycelial clump inoculum, respectively. Furthermore, the ethanol yield was considerably lower at pH 5.5 with the lowest concentration of 0.13 g/g glucose consumed from mycelial clump inoculum. Other fermentation products, especially glycerol, were found to be negligible at lower pH with pellet forms. The addition of spores at pH 3.0 resulted in the formation of small pellets (2.15 ± 0.11 mm) as observed in the previous sections. However, mycelial clump inoculum showed no pellet formation; instead irregularly dispersed short filaments were observed at pH 3.0. In the control test (pH 5.5), inoculation of pellets resulted in mycelial growth around the pellets forming an irregularly shaped biomass clump with a final biomass concertation of 0.29 g dry biomass/g glucose consumed (Fig. 5). Results suggested that low acidic conditions favored glucose uptake and utilization by the fungal pellets. The addition of inoculum as pellets or mycelial clump resulted in higher glucose utilization compared to the spore inoculation at pH 3.0 (Fig. 5).

### Pellet formation in airlift bioreactor

**Fig. 6:**
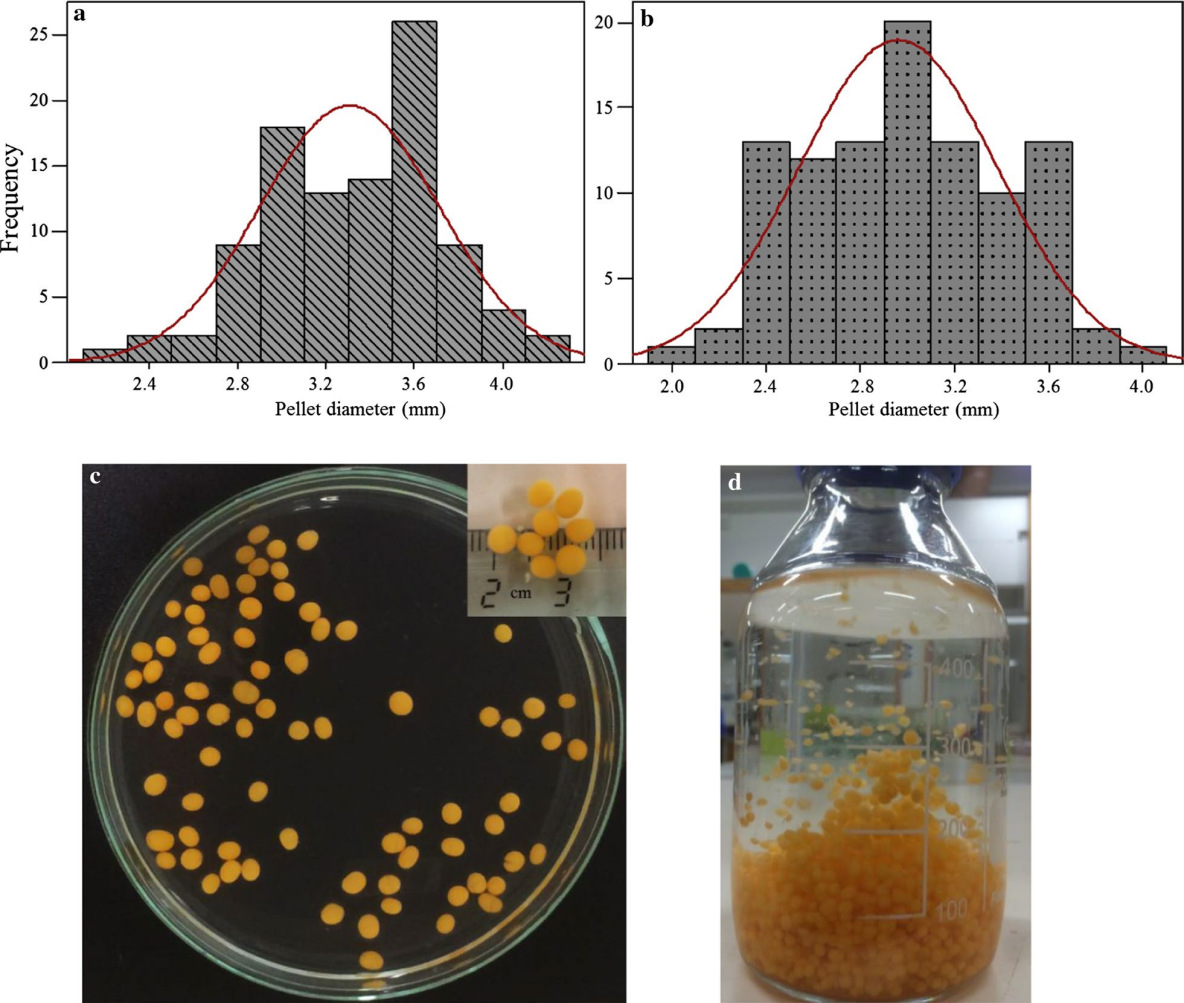
Size distribution of *N. intermedia* pellets formed at culture pH 3 (cultivation for 72 h) in the **a** airlift and **b** bubble column reactors **c** and **d** coalesced fungal pellets from both the reactors

Scale up of the fungal fermentation is preferred in an airlift or bubble column bioreactor as they generally avoid several possible culture limitations instigated by the fungal filaments. Hence, in this study small airlift and bubble column bioreactors (4.5 L) were used to determine the scaling-up potential of the pelletization process. To enhance the fungal biomass production with effective mixing and oxygen transfer, the cultivation in the airlift reactor was carried out at a high aeration condition (1.4 vvm) compared to the lower aeration rate (0.71 vvm) of the bubble column reactor runs. Fungal pellets with consistent shape and size were observed at the end of the cultivation (72 h) with the final pH of the spent medium recorded to be 3.29 ± 0.40 for airlift culture and 2.96 ± 0.42 for bubble column culture (with initial pH 3.0 ± 0.5). Compared to the shake flask, an increased final biomass concentration with 0.298 ± 0.014 and 0.326 ± 0.011 g dry fungal biomass/g glucose consumed was observed while scaling up the pelletization process in the airlift and bubble column bioreactor, respectively. The pellet morphology remained the same in both types of bioreactors, showing no significant influence of the cultivation methods on the pellet formation. The cultivation of fungal pellets in the airlift reactor results in a final cell growth density (packing density) of 50.6 g dry fungal biomass/L cultivation medium compared to the cell density of mycelial filaments (31.3 g dry fungal biomass/L cultivation medium), and with a pellet sedimentation velocity of 1.66 ± 0.2 cm s^−1^ in distilled water, hence proving their potential for high cell densities fermentation cultivation. The average pellet diameter (from both airlift and bubble column cultures together) was measured to be 3.15 ± 0.13 mm, with the size distribution ranging from 2.5 to 4.25 mm (Fig. 6). However, a reduced ethanol production with 0.188 ± 0.004 g and 0.162 ± 0.002 g/g glucose consumed was observed in airlift and bubble column culture respectively, compared to the shake flask cultures. A negligible amount of glycerol was also observed in both the reactor runs with an average of 0.004 ± 0.000 g/g glucose consumed.

## Discussion

Industrial fermentative processes using filamentous fungi have several inherent challenges. Pelletization of filamentous fungi is one of the most studied methods with potential to overcome several of these challenges. The growth of filamentous fungi as pellets in submerged cultures has been described for many different filamentous fungal species such as *Aspergillus*, *Rhizopus* or *Penicillium* strains (Fujita et al. 1994; Liu et al. 2008; Saraswathy and Hallberg 2005; Zhou et al. 2000). In the present study growth of the edible filamentous fungi, *N. intermedia* as pellets is reported for the first time and the factors influencing its formation. Literature reports suggest the influence of multiple factors such as nutrients, pH, agitation, aeration, inoculum level, substrate concentration, polymer additives, the viscosity of the medium, and surface-active agents to influence fungal pellet formation in submerged cultures (Papagianni 2004; Zhang and Zhang 2015).

Of the various factors screened in this study, pH was the only one to significantly influence pellet formation. Factors such as calcium ions (Liu et al. 2013), trace metals (Zhou et al. 2000), agitation rate (Cui et al. 1997) or carbon source (Karmakar et al. 2012), which otherwise influence pellet formation showed no significant effects on *N. intermedia*. Previous studies have revealed that pH is an important factor for pellet formation in most filamentous fungi, but that different strains respond differently to the same pH value. The influence of pH on pellet formation is mainly considered to be through the change in the surface properties of the fungal cells (Metz and Kossen 1977). In the current study, it was observed that low pH conditions, especially the range 3–4 most favored the pellet formation in *N. intermedia*. At pH above 4, mycelial clump formation was observed with a reduced biomass yield at pH 4.5 compared to the control culture (at pH optimum 5.5), which could be attributed to the formation of freely suspended mycelial formation at non-optimum pH conditions (Fig. 1). Similar observations on the influence of cultivation pH on the fungal pellet formation have previously been reported. Zhou et al. (2000) studied the influence of pH on the pellet formation in *Rhizopus oryzae* and reported uniformly distributed, small spherical pellets (<1 mm diameter) at a lower acidic pH range 3.3–2.6.

While studying the influence of agitation rate on *N. intermedia* pellet formation, it was observed that pellets formed at pH 3.0 showed an increased size at the lowest agitation rate (100 rpm) as compared to the pellet formed at higher agitation rates (120 and 150 rpm) (Fig. 4). Similar observation on the influence of agitation rates on fungal pellet size has been reported previously in *R. oryzae* strain (Liu et al. 2008). They demonstrated that low shaking speeds (from 115 to 180 rpm) were beneficial for pellet formation, with pellet size increasing (1.35–4.5 mm) with a decrease in the shaking speed (180–115 rpm). While studying the pellet morphology of *Aspergillus terreus,* Casas López et al. (2005) also made similar observations, where at a lower agitation rate (300 rpm) the pellet size increased as compared to a high-intensity agitation (800 rpm) that forms reduced pellet size. This could be attributed to the variations in the oxygen diffusion efficiency inside the pellets at varying culture conditions. However, data on such investigations are lacking in the case of *N. intermedia* pellets and hence opens the scope for future studies. Interestingly, in the present study, no pellet formation was observed in cultures with different agitation rates at pH 5.5 (control) (Table 1). Hence, it could be concluded that *N. intermedia* pellet formation is initiated only at lower pH conditions irrespective of the agitation rate. Attempts have also been made to determine the effect of inoculum size and type on the process of pellet formation at acidic pH conditions. Inoculum size (spore concentration) is another important factor for pellet formation and previous studies have concluded that low inoculum concentrations are beneficial for pellet production in several strains of filamentous fungi (Liao et al. 2007; Žnidaršič et al. 2000). However, in the present study, no considerable influence of inoculum size or method of inoculation were observed on *N. intermedia* pellet formation.

At all the culture conditions, ethanol and glycerol were the main metabolites formed by the *N. intermedia* pellets. Interestingly a positive correlation between pellet formation and the ethanol production was observed at lower pH conditions. Results suggest that at acidic pH conditions, efficient glucose utilization was observed with pellet cultures as compared to the mycelial clump, resulting in an improved ethanol production (50 % increase). Cultivation of fungal pellets with the sequential glucose addition resulted in about 96–98 % of theoretical ethanol yield (at acidic pH 3.5 and 3.0) as opposed to 32 % theoretical yield obtained by the mycelial clump culture at the same cultivation conditions. The higher ethanol production capabilities of fungal pellets compared to the mycelial clump have not been reported to date, to the best of our knowledge. At lower acidic conditions (especially at pH 3.0 and 3.5) there was only a negligible amount of glycerol being produced, which could be attributed to the lower biomass yield. The growth of fungal pellets at higher cell concentration compared to the mycelial clumps observed in this study possess several advantages in scaling up the process of fungal cultivations at high cell density cultures for various industrial applications using *N. intermedia*. However, an in-depth study is needed to determine the physiological mechanism underlying the effects of acidic pH on the pellet formation.

In conclusion, the present study reports for the first time, the growth of the filamentous ascomycete fungus, *N. intermedia* in the form of pellets. Results suggest that of the several reported factors that generally influence pellet formation in filamentous fungi, pH was observed to be the most influential factor for *N. intermedia* pellet with an initial pH level of 3.0–4.0 strongly supporting pellet formation. The pellet formation remained unaffected by the type of the initial inoculum used, either in the form of fungal spores or mycelial clump or pellets. The pellet size showed an inverse correlation with the agitation rate of the culture, forming larger pellets at lower agitation rate. In a modified fed-batch culture, pellet morphology remained unaffected with sequential glucose addition resulting in a higher final ethanol yield compared to the mycelial clump culture. Scaling-up of the process was achieved in airlift and bubble column bioreactors resulting in the growth of uniform pellets with the size distribution ranging from 2.5 to 4.25 mm. The growth of fungal pellets possesses several advantages in scaling up the fungal cultivations at high cell density cultures, for various industrial applications using *N. intermedia.*
